# Yomogin, Isolated from *Artemisia iwayomogi*, Inhibits Neuroinflammation Stimulated by Lipopolysaccharide via Regulating MAPK Pathway

**DOI:** 10.3390/antiox12010106

**Published:** 2022-12-31

**Authors:** Jin Hee Kim, In Gyoung Ju, Namkwon Kim, Eugene Huh, So-Ri Son, Joon Pyo Hong, Yujin Choi, Dae Sik Jang, Myung Sook Oh

**Affiliations:** 1 Department of Biomedical and Pharmaceutical Science, Kyung Hee University, Seoul 02447, Republic of Korea; 2 Department of Oriental Pharmaceutical Science, Kyung Hee East-West Pharmaceutical Research Institute, College of Pharmacy, Kyung Hee University, Seoul 02447, Republic of Korea

**Keywords:** yomogin, neuroinflammation, p38 mitogen-activated protein kinases, JNK mitogen-activated protein kinases, ERK extracellular signal-regulated kinase, *Artemisia iwayomogi*

## Abstract

Neuroinflammation causes various neurological disorders, including depression and neurodegenerative diseases. Therefore, regulation of neuroinflammation is a promising therapeutic strategy for inflammation-related neurological disorders. This study aimed to investigate whether yomogin, isolated from *Artemisia iwayomogi,* has anti-neuroinflammatory effects. First, we evaluated the effects of yomogin by assessing pro-inflammatory mediators and cytokines in lipopolysaccharide (LPS)-stimulated BV2 microglial cells. The results showed that yomogin inhibited the increase in neuroinflammatory factors, including nitric oxide, inducible nitric oxide synthase, cyclooxygenase-2, interleukin-6, and tumor necrosis factor-α, and suppressed phosphorylation of c-Jun N-terminal kinase, extracellular signal-regulated kinase and p38, which participate in the mitogen-activated protein kinase (MAPK) pathway. To confirm these effects in vivo, we measured the activation of astrocyte and microglia in LPS-injected mouse brains. Results showed that yomogin treatment decreased astrocyte and microglia activations. Collectively, these results suggest that yomogin suppresses neuroinflammation by regulating the MAPK pathway and it could be a potential candidate for inflammation-mediated neurological diseases.

## 1. Introduction

Neuroinflammation, an immune reaction within the central nervous system (CNS), is a response to inflammatory stimulus or neuronal insult [1]. Microglial cells are the major immune cells in the brain and play a pivotal role in the innate immune response [2]. Microglia are activated by inflammatory stimuli, including lipopolysaccharides (LPS), and the activated microglia release various pro-inflammatory molecules and cytokines, including nitric oxide (NO); tumor necrosis factor-α (TNF-α); interleukin-6 (IL-6); and free radicals [3,4]. Excess of these inflammatory factors activates another immune-related cell in CNS, astrocyte. Activated microglia and astrocyte contribute to the development of psychiatric disorders and neurological disorders, such as depression, stroke, and neurodegenerative disease [5,6,7]. Thus, suppression of neuroinflammation can be a therapeutic approach for psychiatric disorders and neurological disorders.

A number of studies have shown that compounds derived from natural products prevent or treat neurological disorders via exerting anti-neuroinflammatory effects [8,9,10]. For example, berberine, a compound isolated from *Coptis chinensis*, was reported to improve depressive behavior by inhibiting pro-inflammatory cytokines such as TNF-α and IL-6 in a mouse depression model caused by chronic unpredictable mild stress [11]. Rosmarinic acid was found to suppress pro-inflammatory cytokines and apoptotic regulators and reduce acetylcholinesterase activity in memory and cognitive impairment models induced by LPS [12]. Fisetin, a natural flavonoid found in various fruits, was reported to reduce inflammatory mediators by inhibiting the nuclear factor kappa B pathway, thereby resulting in amelioration of Alzheimer’s disease (AD) pathology in an amyloid beta injected AD mouse model [13].

In our previous study, we discovered the anti-neuroinflammatory effect of Artemisiae Iwayomogii Herba (AIH) and isolated yomogin as one of the active compounds in the ethyl acetate fraction of AIH exrtacts. Yomogin eudesmane sesquiterpene lactone present in *Artemisia princeps* and *Artemisia vulgaris* [14,15]. Studies have reported that yomogin exerts anticancer properties, whereby it increases caspase-8 activation, with the subsequent release of cytochrome c into the cytoplasm, and inhibits degranulation in basophilic leukemia RBL-2H3 cells. Furthermore, it downregulates inflammatory response by suppressing NO and inducible nitric oxide synthase (iNOS) in LPS-treated macrophage RAW 264.7 cells [16,17,18]. However, the effect of yomogin on the inflammatory response in the CNS has not been studied.

In this study, we aimed to evaluate whether yomogin has an anti-neuroinflammatory effect in LPS-stimulated BV2 microglial cells and to confirm its effect in LPS-injected mice. To estimate the effects of yomogin on LPS-induced NO production without causing cell toxicity, we performed NO and MTT assays. To investigate possible yomogin mechanisms, we measured mRNA and protein levels of iNOS, cyclooxygenase-2 (COX-2), TNF-α, and IL-6, and analyzed the phosphorylation of mitogen-activated protein kinases (MAPKs) in BV2 microglia cells by performing quantitative real-time reverse transcription polymerase chain reaction (qRT-PCR) and Western blotting. Additionally, we evaluated the inhibitory effect of microglia activation in LPS-injected mouse brains by immunofluorescence and performed a forced swimming test (FST) to determine whether yomogin improved the behavior in mice subjected to LPS-induced behavior alteration.

## 2. Materials and Methods

### 2.1. Materials

Dulbecco’s Modified Eagle’s Medium (DMEM), fetal bovine serum (FBS), penicillin-streptomycin were purchased from Hyclone Laboratories, Inc. (Logan, UT, USA). Rabbit antibodies against COX-2, iNOS, c-Jun N-terminal kinase (JNK), phospho-JNK, p38, phospho-p38, extracellular signal-regulated kinase (ERK) and phospho-ERK were purchased from Cell Signaling Technology (Danvers, MA, USA). Rabbit anti-ionized calcium-binding adapter molecule-1 (Iba-1) was purchased from Millipore Bioscience Research (Bedford, MA, USA). Mouse β-actin antibody and goat anti-Glial fibrillary acidic protein (GFAP) were purchased from Santa Cruz Biotechnology (Temecula, CA, USA). Anti-rabbit horseradish peroxidase (HRP) secondary antibodies were purchased from Enzo Life Science, Inc. (Farmingdale, NY, USA). Skim milk was purchased from BD Transduction Laboratories (Franklin Lakes, NJ, USA). Polyvinylidene difluoride (PVDF) was purchased from Millipore (Burlington, MA, USA). Chicken anti-goat Alexa Fluor 488 was purchased from Invitrogen (Carlsbad, CA, USA). 4′,6-diamidino-2-phenylindole (DAPI) were purchased from Vector Laboratories (Burlingame, CA, USA). IL-6 and TNF-α enzyme-linked immunosorbent assay (ELISA) mouse kits were purchased from BD Transduction Laboratories (Franklin Lakes, NJ, USA). Tetramethylethylenediamine, protein assay reagent, acrylamide and enhanced chemiluminescence (ECL) reagent were purchased from Bio-Rad Laboratories (Hercules, CA, USA). Radio-immunoprecipitation assay (RIPA) buffer and protease/phosphatase inhibitor cocktail were obtained from Thermo Fisher Scientific (Waltham, MA, USA). Fluorescent mounting medium was purchased from Dako Cytomation (Glostrup, Denmark). 3-(4,5-Dimethyl-2-thiazolyl)-2,5-diphenyl-2H-tetrazolium bromide (MTT) and all other reagents were purchased from Sigma-Aldrich (St. Louis, MO, USA) unless noted.

### 2.2. Extraction and Isolation of Yomogin

**Figure 1:**
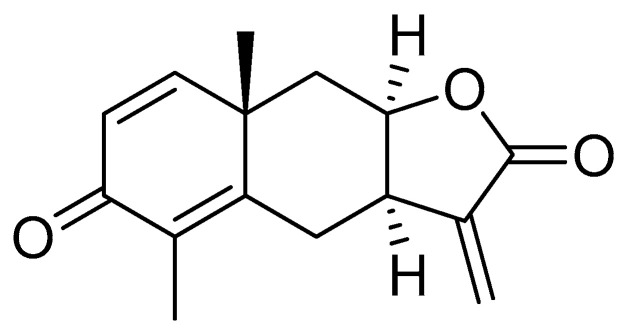
Chemical structure of yomogin.

Yomogin were isolated from AIH according to previously published procedures [19]. The dried AIH (3.0 kg) were extracted twice with 90% ethanol (30 L) 48 h at room temperature, and then the solvent was evaporated in vacuo at 45 °C. The 90% EtOH extract (300.0 g) was suspended in distilled water, and partitioned with ethyl acetate three times. The ethyl acetate fraction (130.0 g) was chromatographed on Diaion HP-20 (*Φ* 11.3 × 55.6 cm) with a gradient system (CH_3_COCH_3_:H_2_O = 30:70 ~ 100:0, *v*/*v*) to obtain 19 subfractions (P1 ~ P19). Finally, yomogin (979.7 mg) was isolated from fraction P7 (8.76 g) by recrystallization in acetone and methanol mixture (1:1, *v*/*v*) at room temperature. The chemical structure of yomogin (Figure 1) was determined by comparison of its ESI-MS data and NMR spectroscopic data (Figures S1–S3) with previously published data [20].

#### Yomogin

White crystal; ESI-MS (positive mode) *m/z* = 245.02 [M + H]^+^ (mass tolerance = 0.5 amu); ^1^H-NMR (500 MHz, CDCl_3_) *δ*_H_ 1.31 (3H, s, H-14), 1.67 (1H, dd, *J* = 15.5, 5.0 Hz, H-9a), 1.94 (3H, s, H-15), 2.27 (1H, dd, *J* = 13.0 Hz, H-6a), 2.42 (1H, dd, *J* = 15.5, 3.0 Hz, H-9b), 2.96 (1H, dd, *J* = 14.5, 7.5 Hz, H-6b), 3.09 (1H, ddd, *J* = 12.0, 7.0, 5.5 Hz, H-7), 4.48 (1H, m, H-8), 5.72 (1H, d, *J* = 1.0 Hz, H-13a), 6.22 (1H, d, *J* = 9.5 Hz, H-2), 6.25 (1H, d, *J* = 1.0 Hz, H-13b), 6.78 (1H, d, *J* = 9.5 Hz, H-1); ^13^C-NMR (125 MHz, CDCl_3_) *δ*_C_ 11.01 (C-15), 25.87 (C-14), 30.15 (C-6), 38.76 (C-10), 39.12 (C-9), 42.09 (C-7), 75.56 (C-8), 122.22 (C-13), 126.59 (C-2), 131.38 (C-4), 140.56 (C-11), 154.67 (C-5), 155.48 (C-1), 169.91 (C-12), 185.82 (C-3).

### 2.3. Cell Culture and Measurement of Cell Viability

BV2 microglial cells were cultured in DMEM supplemented with 10% FBS and 1% penicillin-streptomycin at 37 °C in a 5% CO_2_ humidified incubator. All experiments were performed 24 h after BV2 microglial cell seeding. Cell density of each plate was 3.0 × 104 cells/well for 96-well plates and 5.0 × 105 cells/well for 6-well plates. The BV2 microglial cells were treated by yomogin (0.1, 1, or 10 µM), or quercetin (10 µM) in serum free media for 24 h or 1 h before 23 h of LPS (100 ng/mL) treat. After replacing the culture medium with MTT solution (1 mg/mL), the BV2 microglial cells were incubated for 3 h at 37 °C. Formulated by MTT, the formazan was diluted in dimethyl sulfoxide and measured on a microplate reader at 570 nm.

### 2.4. Measurement of Extracellular NO

The BV2 microglial cells that were pre-treated yomogin (0.1–10 µM) or quercetin (10 µM) for 1 h and were stimulated with LPS (100 ng/mL) for 23 h. NO was measured by microplate reader at 540 nm by mixing supernatant and Griess reagent (1% sulfanilamide, 0.1% naphthylethylenediamine dihydrochloride, 2% phosphoric acid). Sodium nitrite was used as a standard to calculate the nitrite concentration.

### 2.5. Molecular Docking Calculation

The crystal structure of murine iNOS was retrieved from RCSB PDB database (PDB ID: 3E6T, resolution: 2.0 Å). Prior to molecular docking, iNOS protein was prepared by removing ligands and water, followed by adding polar hydrogen atoms. As a ligand, the 3D structure of yomogin was minimized using the force field MMFF94 calculation in Chem3D Pro 14.0 (Cambridge Soft; PerkinElmer Inc., Waltham, MA, USA). Molecular docking calculations were performed with AutoDock Vina and AutoDock Tools 1.5.6 (The Scripps Research Institute, La Jolla, California, United States of America) utilizing the hybrid Lamarckian Genetic Algorithm (LGA). The size of grid box was 30 Ǻ × 30 Ǻ × 30 Ǻ with the spacing of 0.375 Ǻ. The conformation with the lowest energy (RMSD < 1.0) was selected to investigate protein-ligand interaction using Maestro 12.9 software (Schrödinger LLC, New York, NY, USA). PyMOL 2.4.1. (Schrodinger LLC, New York, NY, USA) was used to construct the final 3D protein-ligand complex visualization.

### 2.6. Western Blot Analysis

BV2 microglial cells were harvested after LPS (100 ng/mL) stimulation for 30 min or 23 h in 6-well plates. The BV2 microglial cells were lysed with RIPA buffer containing protease/phosphatase inhibitor cocktail. Proteins were separated by sodium dodecyl sulfate polyacrylamide gel electrophoresis and transferred into PVDF membranes. The membranes were blocked with 5% skim milk or 5% bovine serum albumin (BSA) for 1 h, then incubated with primary antibody diluted in 1% blocking solution overnight. After washing with mixture Tris-buffered saline (10 mM Tris-HCl, 150 mM NaCl, pH 7.5) including 0.1% Tween 20, membrane was replaced with secondary antibody for 1 h. Protein were detected by ECL reagent, and visualization and quantitative assessment of band were measured using Image Lab Software (Bio-Rad, Hercules, CA, USA).

### 2.7. RNA Extraction and qRT-PCR Analysis

BV2 microglial cells were harvested after 23 h of LPS (100 ng/mL) stimulation in 6-well plates. mRNA was extracted from the harvested cells using Hybrid-R™ (GeneAll, Seoul, Republic of Korea). The extracted mRNA was reverse transcribed into cDNA using TOPscript™ RT DryMIX (Enzynomics, Daejeon, Republic of Korea). The cDNA was subjected to qRT-PCR using TOPreal™ qPCR 2X PreMIX (SYBR Green; Enzynomics) and the CFX Connect Real-Time PCR System (Bio-Rad Laboratories, CA, USA). Primers, synthesized at COSMO Genetech (Seoul, Republic of Korea), were as Table 1.

### 2.8. Animals and Treatment

Male ICR (7-week-old) mice were purchased from Daehan Biolink (Eumseong, Republic of Korea). The animals were housed six per cage (size 40 × 25 × 18 cm) with free access to water and food and were kept under constant temperature (23 ± 1 °C) and humidity (60  ±  10%) and a 12 h light/dark cycle. Mice were adapted to their surroundings for 7 days and kept under the same conditions before the start of the study. All animal studies were performed in accordance with the “Guide for the Care and Use of Laboratory Animals, 8th edition” (National Institutes of Health, 2011) and approved by the “Animal Care and Use Guidelines” of Kyung Hee University, Seoul, Republic of Korea (the approval number: KHSASP-20-556).

### 2.9. Experimental Design of Animal Study

Mice were randomly assigned to three groups: Control (*n* = 5), LPS (*n* = 5), LPS + yomogin (*n* = 5). Yomogin liquated in 2% Tween 80 in 1X saline (0.9% NaCl) was administered by oral gavage at 5 mg/kg/day for 3 days. LPS was dissolved in 1x saline and intraperitoneal injection at 5 mg/kg 1 h after the last administration of yomogin. The dosage volume is 1 kg/5 mL. Meanwhile, an equivalent volume of vehicle (1X saline) was given in each group. In addition, the mice were sacrificed immediately after the FST at 3 h after LPS injection.

### 2.10. FST

Each mouse was placed in a glass cylinder (25 cm height × 14 cm diameter) containing 20 cm of water at a temperature of 22 °C. The water was changed between each swim session and the mouse was forced to swim for 6 min. The immobility time was measured by video surveillance during the last 4 min of the 6 min test.

### 2.11. Brain Tissue Preparation

Mice were transcardially perfused with 0.05 M phosphate-buffered saline (PBS), and then fixed with 4% paraformaldehyde in 0.1 M phosphate buffer. Brains were obtained, post-fixed overnight at 4 °C, and then immersed in a solution containing 30% sucrose in PBS for cryoprotection. Serial 30 µm-thick coronal sections were cut on a freezing sliding microtome (Leica Microsystems Inc., Nussloch, Germany) and stored in cryoprotectant (25% ethylene glycol, 25% glycerol, and 0.05 M phosphate buffer) at 4 °C until use.

### 2.12. Immunofluorescence Staining

Brain sections were rinsed in PBS and then replaced with anti-Iba-1 antibody (1:1000 dilution) or anti-GFAP antibody (1:1000 dilution) for overnight at 4 °C containing blocking buffer (0.3% triton X-100, 1% normal chicken serum and 0.03% BSA in PBS). After rinsing in PBS, for visualization, the primary antibody was developed by incubating with Alexa Fluor 488 or 594-conjugated secondary antibodies for 1 h at room temperature. Images were captured with an Olympus BX51 microscope (Olympus, Tokyo, Japan). The areas of Iba-1 and GFAP positive cells in the hippocampus was analyzed with Image J software.

### 2.13. Statistical Analysis

Values are expressed as the mean ± standard error of the mean (S.E.M.) and were produced using Graph Pad Prism 5.0 software (Graph Pad software Inc., San Diego, CA, USA). The results were analyzed statistically by one-way analysis of variance (ANOVA) followed by Tukey’s post hoc test. Differences with a *p* value less than 0.05 were considered statistically significant.

## 3. Results

### 3.1. Effects of Yomogin on Cell Viability and NO Production in LPS-Stimulated BV2 Microglial Cells

**Figure 2:**
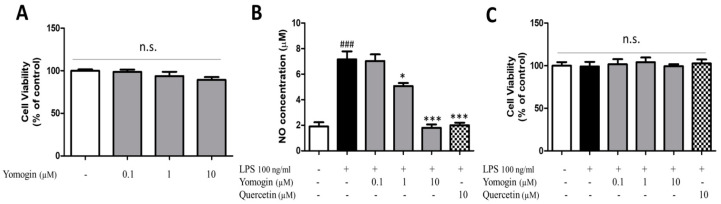
Effects of yomogin on cell viability and NO production in LPS-stimulated BV2 microglial cells. Cells were incubated with yomogin for 24 h without (**A**) or with (**C**) LPS treatment for the last 23 h (*n* = 6 per group). The supernatant containing NO was determined by Griess reagent colorimetric reaction (**B**) (*n* = 6 per group). Quercetin was used as a positive control. Data were analyzed by one-way ANOVA, followed by Tukey’s post hoc test. ### *p* < 0.001 compared to the control group; * *p* < 0.05 and *** *p* < 0.001 compared to the LPS only treated group. Values are expressed as mean ± SEM.

To assess the cytotoxicity of yomogin in BV2 cells, cells were treated with yomogin at various doses (0.1–10 μM). There was no cytotoxicity in the cells cultured in presence of yomogin (Figure 2A). We measured the concentration of NO in the supernatant of LPS-stimulated BV2 microglial cells to assess the effect of yomogin on inflammatory suppression. We found that LPS stimulation elevated NO levels while yomogin at 1 μM and 10 μM decreased NO levels significantly compared to the LPS-only-treated group without cytotoxicity (Figure 2B,C). Moreover, the suppressive effect of 10 μM yomogin on NO production was similar to that of quercetin.

### 3.2. Molecular Docking Results of Yomogin and iNOS Enzymes

**Figure 3:**
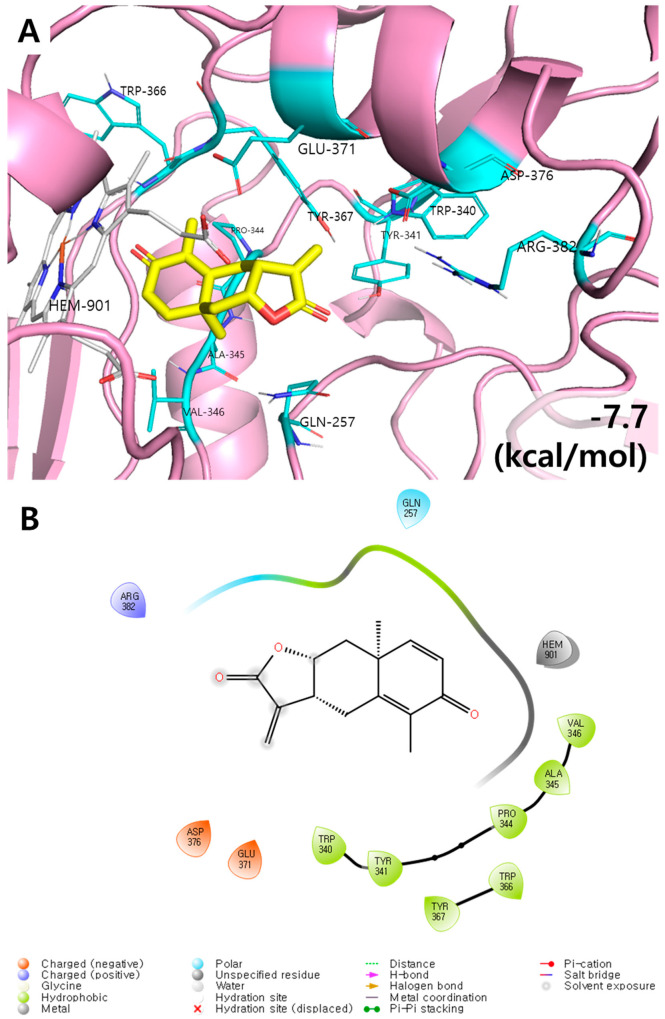
Molecular docking results of yomogin and iNOS enzyme. The 3D (**A**) and 2D (**B**) diagrams of iNOS and yomogin molecular docking simulations were determined at the lowest energy conformation (RMSD < 1.0) Each atom is colored as follows: red for oxygen, blue for nitrogen, and yellow/cyan for carbon. Only the interacting residues are labeled for clarity. PyMOL and Maestro were used to generate graphical representations of the interactions.

One of three types of NOS proteins, iNOS is a key protein in the synthesis of NO along with L-arginine and cofactors [21]. To investigate the inhibitory effect of yomogin on NO synthesis, the molecular docking calculation was conducted between iNOS and yomogin. The lowest energy conformation showed that yomogin has the good binding affinity with iNOS (−7.7 kcal/mol, RMSD < 1.0). Hydrophobic pocket was observed in this conformation with residues TRP340–VAL346 and TRP366–TYR367, suggesting that they contribute in iNOS binding via non-hydrogen bonding interaction. Two negative charge interactions with GLU371 and ASP376 as well as one positive charge interaction with ARG382 were observed. A polar interaction with residue GLN257 were also found in the present simulation (Figure 3). This finding indicated that yomogin inhibits the NO production by binding to the iNOS protein.

### 3.3. Effects of Yomogin on Expressions of iNOS and COX-2 in LPS-Stimulated BV2 Microglial Cells

**Figure 4:**
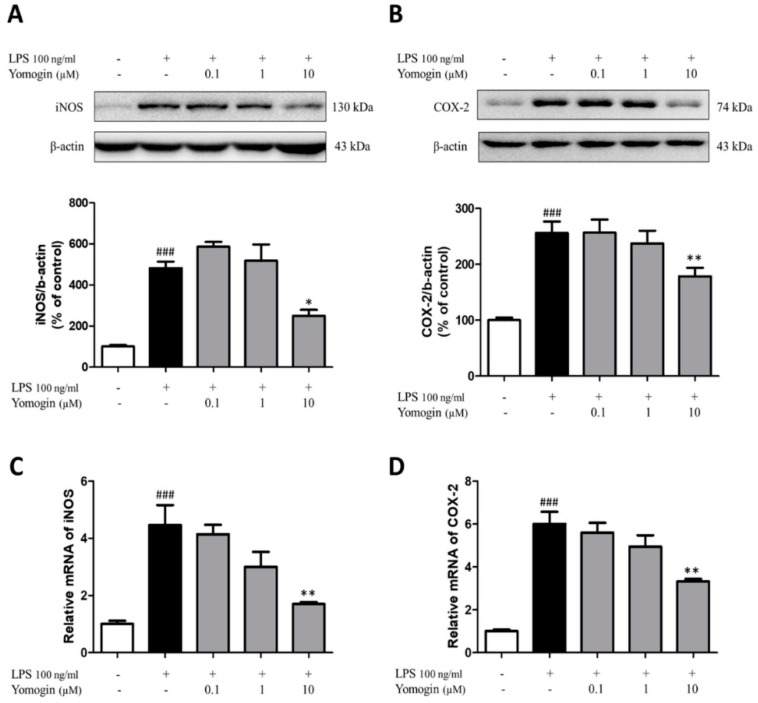
Effects of yomogin on expressions of iNOS and COX-2 in LPS-stimulated BV2 microglial cells. The cells were treated with various concentrations (0.1–10 µM) of yomogin for 1 h before LPS treatment. The protein levels of iNOS (**A**) and COX-2 (**B**) were determined by Western blotting (*n* = 5–6 per group). The mRNA levels of iNOS (**C**) and COX-2 (**D**) were analyzed by qRT-PCR (*n* = 6 per group). Data were analyzed by one-way ANOVA, followed by Tukey’s post hoc test. ### *p* < 0.001 compared to the control group; * *p* < 0.05 and ** *p* < 0.01 compared to the LPS-only-treated group. Values are the mean ± SEM.

iNOS and COX-2 are reported to produce neurotoxic factors such as NO in the CNS [1]. We observed that yomogin treatment at 10 µM significantly inhibited LPS-stimulated iNOS and COX-2 mRNA levels. Additionally, iNOS and COX-2 protein levels induced by LPS were suppressed by yomogin treatment at 10 µM (Figure 4). These results suggest that the suppression of NO in Figure 2 is due to the decrease in iNOS expression.

### 3.4. Effects of Yomogin on Secretion of TNF-α and IL-6 in LPS-Stimulated BV2 Microglial Cells

**Figure 5:**
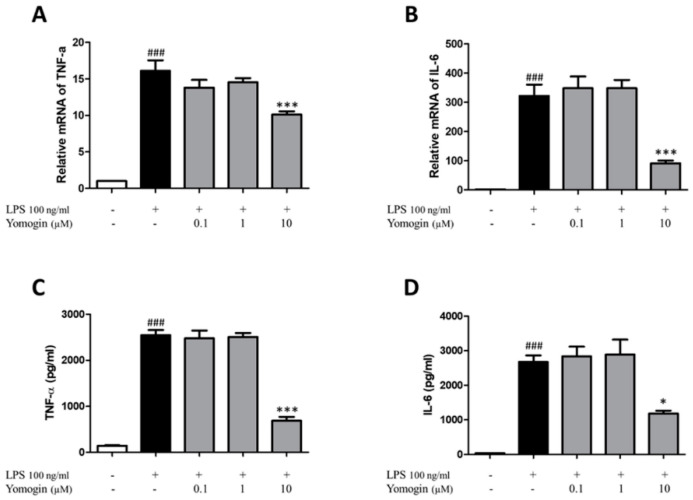
Effects of yomogin on secretion of TNF-α and IL-6 in LPS-stimulated BV2 microglial cells. The cells were pre-treated various concentrations (0.1–10 µM) of yomogin for 1 h before LPS treatment. The mRNA levels of TNF-α (**A**) and IL-6 (**B**) were measured by qRT-PCR (*n* = 4). The levels of TNF-α (**C**) and IL-6 (**D**) were analyzed in the supernatants by ELISA kit (*n* = 4 per group). Data were analyzed by one-way ANOVA, followed by Tukey’s post hoc test. ### *p* < 0.001 compared to the control group; * *p* < 0.05 and *** *p* < 0.001 compared to the LPS-only-treated group. Values are the mean ± SEM.

TNF-α and IL-6 are inflammatory cytokines that contribute to the upregulation of inflammatory reactions [22]. We measured the mRNA levels of TNF-α and IL-6. The LPS-only-treated group showed increased TNF-α and IL-6 mRNA levels compared to the control group. However, the levels were significantly lower in cells treated with 10 µM yomogin than in the LPS-only-treated group (Figure 5A,B). To confirm the mRNA expression results, we performed ELISA to determine whether yomogin inhibited the release of inflammatory cytokines in the supernatant. As shown in Figure 5C,D, TNF-α and IL-6 concentrations increased in the LPS-only-treated group and significantly decreased in yomogin-treated with 10 µM group. This data shows that yomogin exerts anti-inflammatory effects by inhibiting cytokines.

### 3.5. Effects of Yomogin on MAPK Signaling Pathway in LPS-Stimulated BV2 Microglial Cells

**Figure 6:**
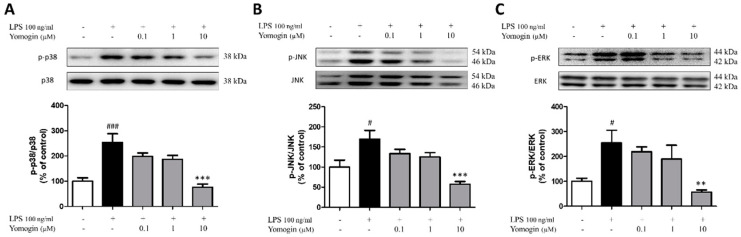
Effects of yomogin on MAPK signaling pathway in LPS-stimulated BV2 microglial cells. Cells were pre-treated with various concentrations (0.1–10 µM) of yomogin for 1 h before LPS treatment. The protein levels of p-p38, p38 (**A**), p-JNK, JNK (**B**) and p-ERK, ERK (**C**) were examined by Western blot analysis (*n* = 4–6 per group). Data were analyzed by one-way ANOVA, followed by Tukey’s post hoc test. # *p* < 0.05 and ### *p* < 0.001 compared to the control group; ** *p* < 0.01, *** *p* < 0.001 compared to the LPS-only-treated group. Values are the mean ± SEM.

We investigated the MAPK signaling pathway that controls inflammatory responses as an upstream regulator of inflammation. We measured the ratios of p38, JNK, and ERK in LPS-stimulated BV2 cells. As shown in Figure 6, the LPS-only-treated group showed an increased ratio of phosphorylated MAPK compared to the control group, whereas the ratios of p38, JNK and ERK were significantly reduced in cells treated with 10 µM yomogin (Figure 6). This data shows that yomogin regulates the downstream factors of MAPK signaling by inhibiting the phosphorylation of p38, JNK and ERK.

### 3.6. Effects of Yomogin on Microglia and Astrocyte Activation in LPS-Treated Mouse Brain

**Figure 7:**
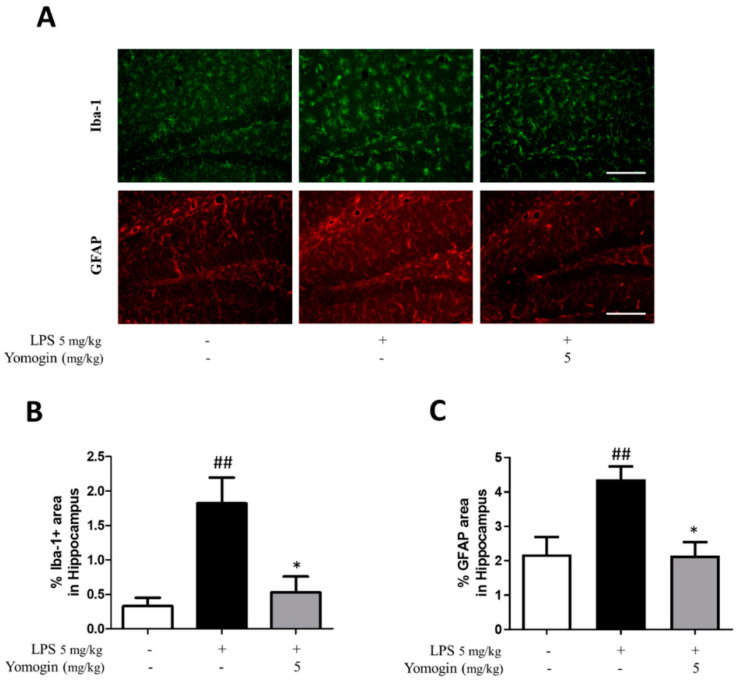
Effects of yomogin on microglia and astrocyte activations in LPS-treated mouse brain. The mice were orally pretreated with yomogin at 5 mg/kg once daily for 3 days. LPS was injected 1 h after administering yomogin, and mice were sacrificed 3 h later. Representative images are shown for the Iba-1 and GFAP positive cells in the hippocampus (**A**). Scale bar = 100 µm. Quantification of Iba-1 (**B**) and GFAP (**C**) positive cells in hippocampus. Data were analyzed by one-way ANOVA, followed by Tukey’s post hoc test. ## *p* < 0.01 compared to the control group; * *p* < 0.05 compared to the LPS-injected group. Values are the mean ± SEM.

Next, we investigated the impact of yomogin on microglial and astrocyte activation in vivo. We performed immunofluorescence using Iba-1 antibody to detect activated microglia. The LPS-injected group showed an increased Iba-1-positive area compared to the control group in the hippocampus and cortex. However, the proportion of Iba-1-positive area was significantly decreased in the yomogin-treated group compared to the LPS group in the same region (Figure 7). In addition, astrocyte activation was measured by GFAP antibody used as a marker for astrogliosis. This data suggests that yomogin inhibits LPS-induced microgliosis and astrogliosis in the mouse brain.

### 3.7. Effects of Yomogin on Depressive Behavior in LPS-Treated Mouse

**Figure 8:**
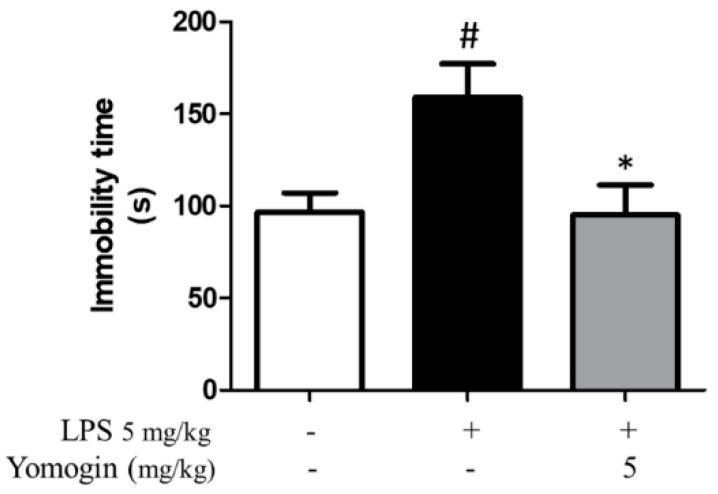
Effects of yomogin on behavioral dysfunction in LPS-treated mouse. Mice were orally pretreated with yomogin at 5 mg/kg once daily for 3 days. LPS was injected 1 h after administering yomogin, and mice were sacrificed 3 h later. For FST analysis, immobility time in water was measured during 4 min (*n* = 5 per group). Data were analyzed by one-way ANOVA, followed by Tukey’s post hoc test. # *p* < 0.05 compared to the control group; * *p* < 0.05 compared to the LPS-injected group. Values are the mean ± SEM.

Neuroinflammation causes depression by disrupting the neurotransmitter system [23]. Accordingly, to measure the effect of yomogin on alleviating depressive behavior induced by LPS stimulation, we performed the FST. In the FST, the immobility time significantly increased in the LPS-injected group compared to the control group, whereas in the yomogin-administered group, the immobility time was significantly lower than that in the LPS-injected group (Figure 8). This result suggests that treatment with yomogin can improve depression induced by LPS.

## 4. Discussion

This study investigated whether yomogin has inhibitory effects on LPS-induced neuroinflammation in silico, in vitro and in vivo. By measuring pro-inflammatory mediators including iNOS, COX-2, and cytokines in BV2 microglial cells, we showed that yomogin effectively regulates these factors. Additionally, we confirmed its effects determined from the in vitro results, which showed that yomogin administration inhibited microglia activation and improved depressive-like behavior in LPS-injected mice.

LPS is a well-known inducer of neuroinflammation in microglial cells [24]. LPS is recognized by Toll-like receptor 4, a widely expressed receptor in microglia that regulates immune responses and activates the MAPK pathway, leading to the production of pro-inflammatory mediators [25,26]. In this study, we showed that yomogin controls the phosphorylation of p38, JNK and ERK. This inhibition of the MAPK signaling pathway results in the reduction of the pro-inflammatory mediators and inducible enzymes, thereby decreasing the NO and cytokine levels.

MAPK signaling plays important roles in depression-regulating mechanisms [27]. Recently, it was found that SB203580, a p38-specific inhibitor, ameliorated depressive-like behaviors and normalized alterations in TNF-α levels in an LPS-injected rat model [28]. Based on these observations, p38 has been proposed as a new target for neuroinflammation-induced depression [29]. Likewise, JNK has emerged as a potential target in depression [30,31]. SP600125, a JNK inhibitor, reduced LPS-induced increase in the levels of TNF-α and interleukin 1β in LPS-injected rat model. Moreover, LPS-induced depressive-like behaviors in rats were alleviated in response to treatment with SP600125 [32]. In this study, yomogin effectively decreased LPS-induced phosphorylation of p38 and JNK in microglia. Therefore, we suggest that yomogin can ameliorate depressive disorders induced by neuroinflammation.

Further, our results confirmed the anti-neuroinflammatory effect of yomogin in LPS-injected mice, a model of neuroinflammation that exhibits various neuropathological symptoms [33]. Furthermore, yomogin inhibited microglial cells activation induced by LPS in the hippocampus and cortex of mouse brains. Additionally, yomogin alleviated depressive-like behavior as observed in the results of FST. Under conditions of brain inflammation, microglial cells produce inflammatory mediators that may influence brain neurotransmitter systems and neuron conditions [34]. Pro-inflammatory cytokines activate inflammatory signaling pathways, leading to changes in neurotransmitters such as glutamate, dopamine, serotonin, and norepinephrine [35]. These alterations are known to cause a variety of neurological disorders, including depression, anxiety, and dementia [36,37]. In this study, we suggest that yomogin suppresses inflammation-related depression as it reduces microglia activation and decreases immobility time in the FST.

Since LPS was injected intraperitoneally, it is unclear whether yomogin affected the brain. Moreover, no study so far has reported that yomogin can pass through the blood–brain barrier (BBB). However, the above results indicated that yomogin exerts beneficial effects on the brain. A previous study revealed that fat-soluble substances with a molecular weight of less than 500 Da are advantageous to the BBB by transmembrane diffusion [38]. Since sesquiterpene lactone is lipophilic and its molecular weight is less than 500 Da, there is a high probability that yomogin would pass through the BBB [39]. Additionally, the possibility of yomogin penetrating the BBB was supported by using the Boiled-Egg algorithm of Swiss ADME calculations. Furthermore, previous studies revealed that BBB is disrupted by signaling changes and enhanced cellular traffic under inflammation condition [40,41]. Therefore, injection of LPS increases the probability of yomogin passing through the BBB in mice. Thus, yomogin might have directly affected the brain by crossing the BBB, thereby reducing microglia and astrocyte activations in the brain.

## 5. Conclusions

In conclusion, this study demonstrated that yomogin suppresses LPS-induced neuroinflammation in microglial cells and mice. These anti-neuroinflammatory effects can be applicable for treating neurodegenerative diseases or psychiatric disorders. Further research is warranted to assess the effects of yomogin on brain disorders or diseases and reveal the underlying mechanism.

## Figures and Tables

**Table 1:** Oligonucleotide sequences used in qRT-PCR experiments.

Gene Symbol	Primer Sequence	Accession Number
iNOS	5′-GTG TTC TTT GCT TCC ATG CT-3′ 5′- AGT TGC TCC TCT TCC AAG GT-3′	NM_001313922.1
COX-2	5′-TGG GGT GAT GAG CAA CTA TT-3′ 5′- AAG GAG CTC TGG GTC AAA CT-3′	NM_011198.4
TNF-α	5′-GAT TAT GGC TCA GGG TCC AA-3′ 5′- GCT CCA GTG AAT TCG GAA AG-3′	NM_001278601.1
IL-6	5′-CCG GAG AGG AGA CTT CAC AG-3′ 5′- TTG CCA TTG CAC AAC TCT TT-3′	NM_001314054.1

## Data Availability

Data Availability Statement: Data is contained within the article and Supplementary Material.

## Supplementary Materials

The following supporting information can be downloaded at: https://www.mdpi.com/article/10.3390/antiox12010106/s1, Figure S1: ^1^H-NMR spectrum of yomogin (500 MHz, chloroform-d); Figure S2: ^13^C-NMR spectrum of yomogin (125 MHz, chloroform-d); Figure S3: ESI-MS data of yomogin.
