# Supplementary material for: Yomogin, Isolated from Artemisia iwayomogi, Inhibits Neuroinflammation Stimulated by Lipopolysaccharide via Regulating MAPK Pathway
Source: Antioxidants (Basel). 2022 Dec 31;12(1):106. doi: 10.3390/antiox12010106 (PMC9854746; doi:10.3390/antiox12010106)
Supplement: Supplementary file 1 [file antioxidants-12-00106-s001.zip › antioxidants-2111298-supplementary.pdf]

Figure S1. ^{1}H-NMR spectrum of yomogin (500MHz, chloroform-*d*).

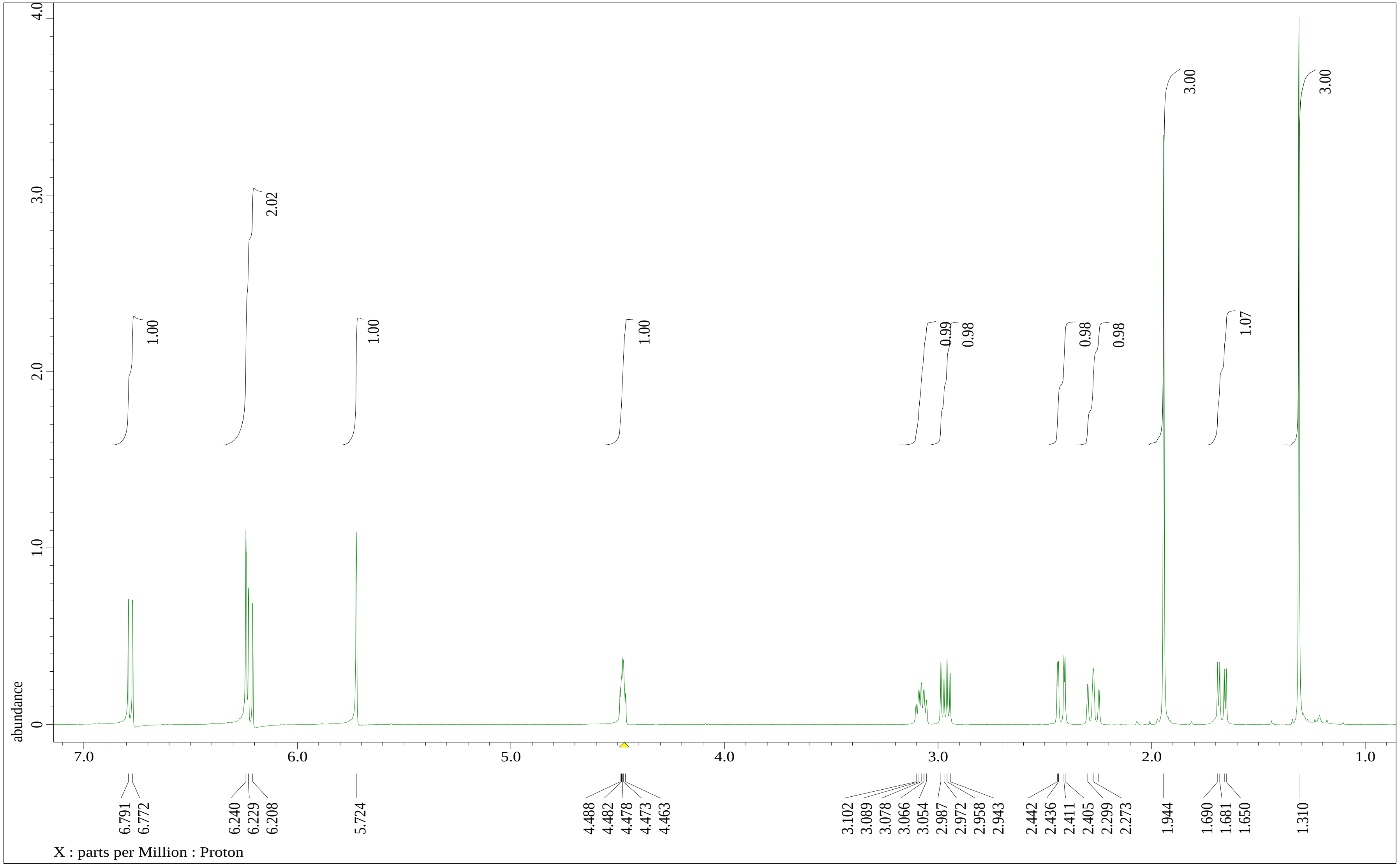

Figure S2. ^{13}C-NMR spectrum of yomogin (125MHz, chloroform-*d*).

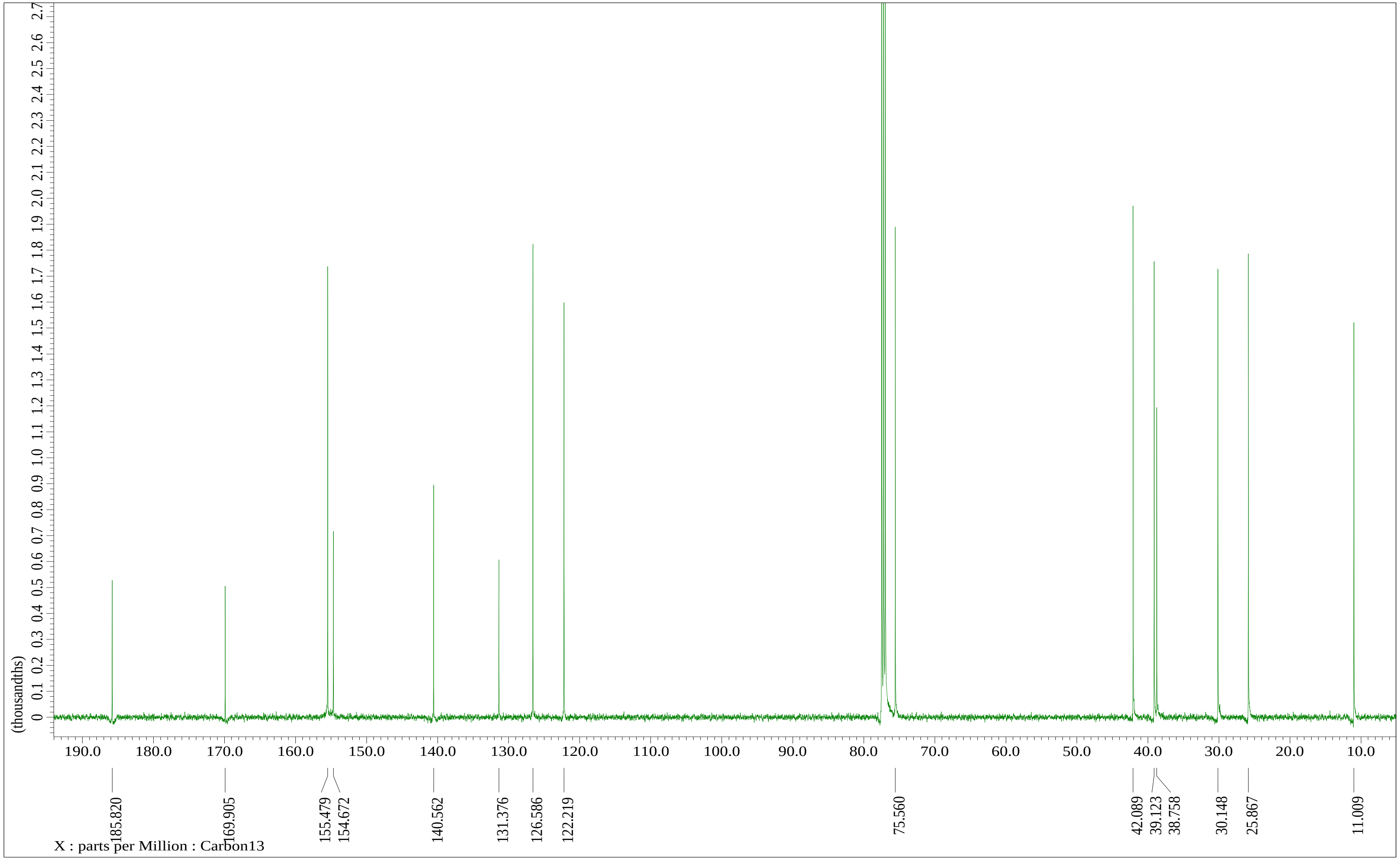

Figure S3. ESI-MS data of yomogin.

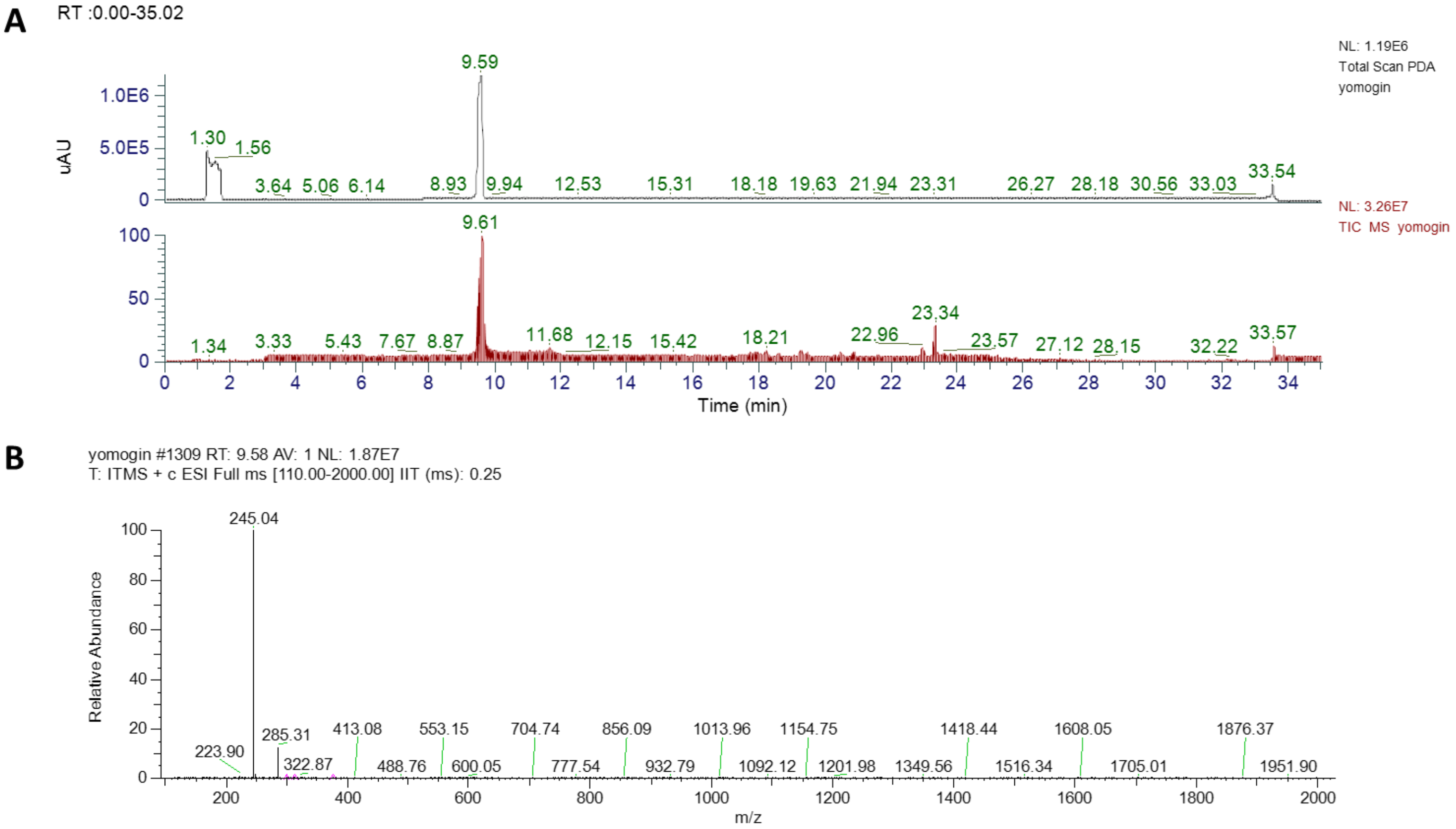

A: UHPLC chromatogram of yomogin at PDA (up, RT: 9.59 min) and total ion chromatogram (TIC) of yomogin (down); **B**: Electrospray ionization (ESI) full-scan precursor ion mass data (positive mode, *m/z* = 245.04, mass tolerance = 0.5 amu). Data collected by Thermo Fisher Vanquish UHPLC and LTQ-XL MS^n system.
